# Supplementary material for: Low rank adaptation of chemical foundation models generates effective odorant representations
Source: bioRxiv. 2026 May 10:2025.11.04.686628. Preprint. [Version 4] doi: 10.1101/2025.11.04.686628 (PMC13105091; doi:10.1101/2025.11.04.686628)
Supplement: Supplement 1 [file media-1.pdf]

Supplement for the paper

Low Rank Adaptation of Chemical Foundation Models Generate Effective Odorant Representations

November 2025

Table 1: Distribution of responsive vs. non-responsive classes in the M2OR dataset.

M2OR Dataset	Responsive	Non-Responsive	% Responsive
EC50 (highest quality)	1,153	4,062	28.4%
Secondary screening (middle)	283	7,875	3.6%
Primary screening (lowest)	1,464	31,726	4.6%
All Data	2,900	43,663	6.6%

Table 2: Predictive performance using a protein-only model. For each test dataset, the model uses only protein features for prediction. Values show mean ± standard deviation across five cross-validation folds. R^2: coefficient of determination, MCC: Matthews correlation coefficient.

Dataset	Protein-Only Prediction
Hallem Dataset (R^2)	-3.56 ± 24.1
Carey Dataset (R^2)	-0.336 ± 1.60
M2OR (EC50) (MCC)	0.098 ± 0.307

For the M2OR (EC50) dataset, odorants with fewer than 23 proteins were excluded from training, due to limited data.

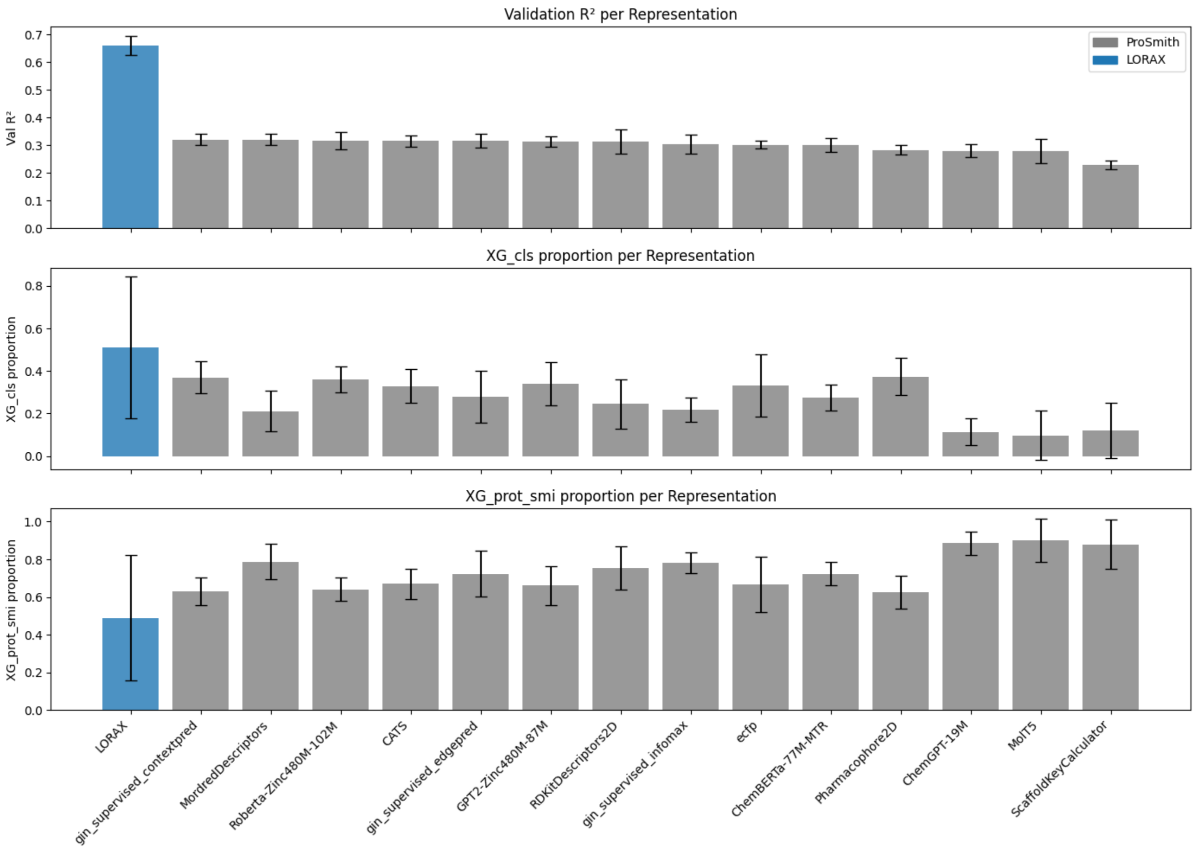

Figure 1: Comparison of LORAX and ProSmith representations after training two XGBoost models on the Carey dataset: one using only the `<cls>` token, and one using the original chemical and protein foundation model representations. (Top) Mean validation R^2 scores for ProSmith and LORAX prior to applying XGBoost. (Middle) The mean weight assigned to the XGBoost model utilizing only the `<cls>` token. (Bottom) The mean weight assigned to the XGBoost model utilizing only the original chemical and protein foundation model representations. All error bars indicate standard deviation across 5-fold cross validation of the data.

Table 3: Hyperparameter sweep of rank (r) of LoRA adapter for the LORAX model. The model was trained on the Carey dataset. Reported values are test set R^2 across folds without the XGBoost ensemble. Bolded value indicates highest performance. All models were trained with query, key, and value weight matrices being adapted.

Rank	rand_split_1	rand_split_2	rand_split_3	rand_split_4	rand_split_5	avg	std
$r = 2$	0.6456	0.5921	0.5045	0.6029	0.6134	**0.5917**	0.0471
$r = 4$	0.6179	0.5802	0.5009	0.5884	0.5621	0.5699	0.0389
$r = 8$	0.6267	0.6115	0.4986	0.5892	0.6082	0.5868	0.0457
$r = 16$	0.6122	0.5965	0.5183	0.5506	0.6212	0.5798	0.0392

Table 4: Hyperparameter sweep of rank LoRA adapted modules for the LORAX model. The model was trained on the Carey dataset. Reported values are test set R^2 across folds without the XGBoost ensemble. Bolded value indicates highest performance.W_q, W_k, and W_v, indicate query, key, and value weight matrices respectively. All models were trained with rank 8 adapter matrices.

Adapted Modules	rand_split_1	rand_split_2	rand_split_3	rand_split_4	rand_split_5	avg	std
W_q	0.6426	0.6148	0.5505	0.5727	0.6018	**0.5964**	0.0322
W_q, W_k	0.6437	0.6113	0.5162	0.5674	0.6085	0.5894	0.0439
W_q, W_v	0.6312	0.5993	0.5446	0.5700	0.5908	0.5872	0.0290
W_q, W_k, W_v	0.6267	0.6115	0.4986	0.5892	0.6082	0.5868	0.0457

Table 5: Pairwise post hoc comparisons between models for unseen odorants (as presented in Table 2, main text). Results are based on Conover's signed-rank test following a significant Friedman test; all p-values were corrected for multiple comparisons using the Benjamini-Hochberg false discovery rate (FDR) procedure.

		T-Stat	df	W_i	W_j	r_{rb}	p	p_{FDR}
LORAX	ProSmith	2.353	8	14.000	8.000	1.000	.046	.069
	Naive	2.353	8	14.000	8.000	0.467	.046	.069
ProSmith	Naive	0.000	8	8.000	8.000	0.067	1.000	1.000

Table 6: Pairwise post hoc comparisons between models for unseen receptors (as presented in Table 2, main text). Results are based on Conover's signed-rank test following a significant Friedman test; all p-values were corrected for multiple comparisons using the Benjamini-Hochberg false discovery rate (FDR) procedure.

		T-Stat	df	W_i	W_j	r_{rb}	p	p_{FDR}
LORAX	ProSmith	0.000	8	12.00	12.000	−0.067	1.000	1.000
	Naive	2.353	8	12.00	6.000	1.000	.046	.069
ProSmith	Naive	2.353	8	12.00	6.000	0.867	.046	.069

Table 7: Pairwise post hoc comparisons between Matthews correlation coefficient (MCC) values for LORAX and ProSmith models on M2OR in Table 3 of the main text. Results are based on Conover's signed-rank test following a significant Friedman test; all p-values were corrected for multiple comparisons using the Benjamini-Hochberg false discovery rate (FDR) procedure.

		T-Stat	df	W_i	W_j	r_{rb}	p	p_{FDR}
LORAX	ProSmith	2.121	8	14.00	11.000	0.733	.067	0.067
	MolOR (Weighted)	6.364	8	14.00	5.000	1.000	$< .001$	< 0.003
ProSmith	MolOR (Weighted)	4.243	8	11.00	5.000	1.000	.003	0.0045

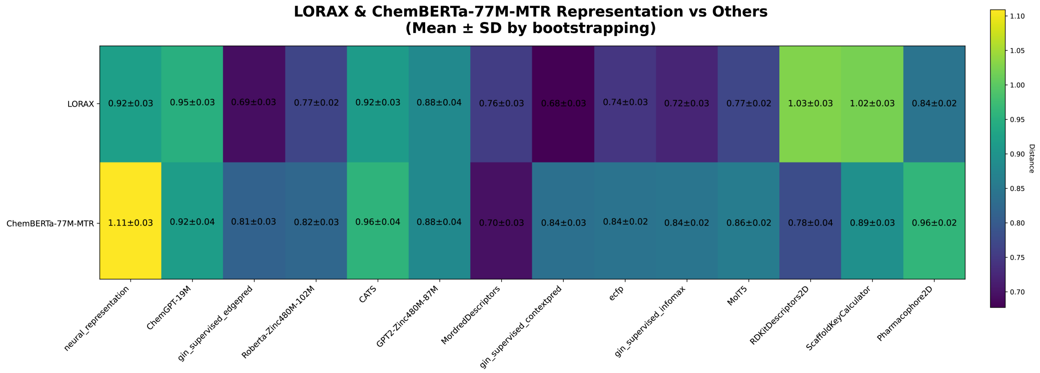

Figure 2: CCA distance matrix for LORAX and ChemBERTa-77M-MTR representations on the Carey dataset, estimated via 1,000 bootstraped samples. Each cell reports the mean ± standard deviation of distances computed over the 1,000 smaples. For each bootstrap iteration, 110 odorants were randomly selected with replacement, and CCA distances were computed between the corresponding feature spaces. Bootstrapped CCA distances between LORAX and ChemBERTa-77M-MTR representations and all other models were compared using Welch's t-tests, with multiple comparisons controlled via false discovery rate (FDR) correction. All pairwise differences were statistically significant ($p < 0.0001$), except for those involving GPT2-Zinc480M-87M.

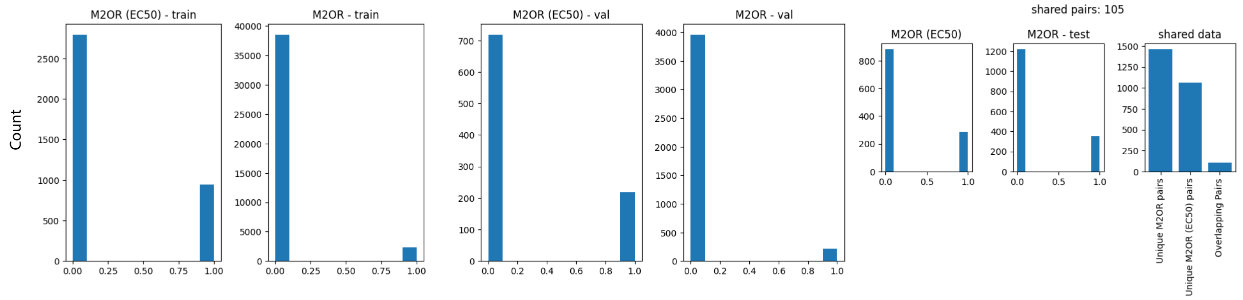

Figure 3: Distributions of train, validation, and test splits for split 1 in both the M2OR and M2OR (EC50) datasets. Although the test sets share similar overall distributions, substantial differences are observed in the train and validation sets. Furthermore, the test set composition varies considerably between the two datasets.

Table 8: Comparison of various LORAX models trained on the M2OR dataset. LORAX (C) and LORAX (P + C) are taken from the main text Table 3. LORAX (U) uses Uni-Mol2-84M (Ji et al., 2024) as the chemical foundation model instead of ChemBERTa-77M-MTR. Uncertainties for all models are standard deviation across 5 fold cross validation. Bold indicates highest value in each column. n/a entries indicate that the authors did not report that metric. AUROC: area under the receiver operating characteristic curve, AveP: average precision, MCC: Matthews correlation coefficient.

	AUROC	AveP	Precision	Recall	F-score	MCC
LORAX (U)	80.36 ± 1.02	0.775 ± 0.03	**0.732 ± 0.05**	0.679 ± 0.02	0.703 ± 0.02	0.624 ± 0.03
LORAX (P + C)	82.43 ± 0.86	0.776 ± 0.03	0.729 ± 0.06	0.727 ± 0.03	0.727 ± 0.02	0.650 ± 0.03
LORAX (C)	**83.24 ± 1.34**	**0.778 ± 0.03**	0.710 ± 0.05	**0.754 ± 0.02**	**0.730 ± 0.03**	**0.651 ± 0.04**

Table 9: Performance of LORAX and a Naïve Baseline on Hallem and M2OR (EC50) Datasets for Unseen Odorants and Receptors. Metrics for each split are reported in R^2 for the Hallem dataset and Matthews Correlation Coefficient (MCC) for the M2OR (EC50) dataset. For the Naïve baselines, predictions for the Hallem dataset were obtained by calculating the mean of the training set, and using that value as a guess for every test example. For the M2OR (EC50) dataset, Naïve predictions were generated by randomly assigning either 0 or 1 to each test example with equal probability.

Dataset	Scenario	Method	rand_split_1	rand_split_2	rand_split_3	rand_split_4	rand_split_5	avg	std
Hallem	Unseen odorants	LORAX	-0.599	-1.138	0.250	0.292	0.421	**-0.155**	0.610
		Naïve	-0.499	-0.394	-0.003	-0.102	-0.076	-0.215	0.195
Hallem	Unseen receptors	LORAX	0.018	-0.021	0.087	0.151	-2.358	-0.425	0.968
		Naïve	-0.049	-0.812	-0.001	-0.087	-0.052	**-0.200**	0.307
M2OR (EC50)	Unseen odorants	LORAX	0.634	0.736	0.522	0.622	0.310	**0.565**	0.144
		Naïve	0.078	-0.030	-0.041	0.078	-0.033	0.010	0.055
M2OR (EC50)	Unseen receptors	LORAX	0.351	0.295	0.389	0.149	0.244	**0.286**	0.0841
		Naïve	-0.043	-0.037	-0.005	-0.010	0.023	-0.014	0.024
